# Correction: Silva, P.J. Computational Development of Inhibitors of Plasmid-Borne Bacterial Dihydrofolate Reductase. *Antibiotics* 2022, *11*, 779

**DOI:** 10.3390/antibiotics13111041

**Published:** 2024-11-04

**Authors:** Pedro J. Silva

**Affiliations:** 1 FP-I3ID, FP-BHS, Faculdade de Ciências da Saúde, Universidade Fernando Pessoa, 4200-150 Porto, Portugal; pedros@ufp.edu.pt; 2 UCIBIO@REQUIMTE, BioSIM, Departamento de Biomedicina, Faculdade de Medicina, Universidade do Porto, 4200-319 Porto, Portugal

## Error in Figure and Table

In the original publication [1], there was a mistake in Figure 1 as published. The depictions of molecules **31** and **32** in Figure 1 erroneously contained a 2H-pyran ring instead of the correct 1,2-dihydropyridine ring. The corrected portion of Figure 1 appears as follows:

**Figure:**
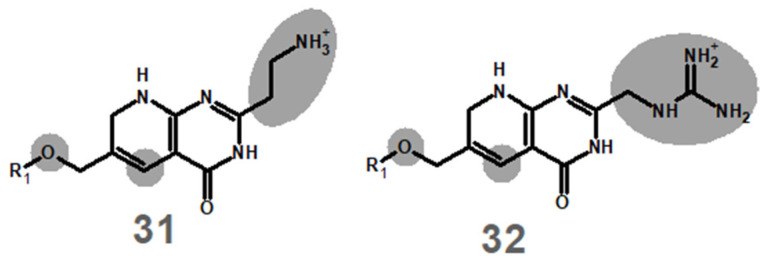


The protonation and reduction energies given in the original Table 1 referred to the 2H-pyran-containing analogues. The correct energies for the actual molecules **31** and **32** are given in Table 1 below. I apologize to the readers for this oversight. The results and conclusions of the original publication are not affected, since the protonation and reduction energies of these molecules are confirmed to render both its protonation and its reduction by NADH unfeasible, and therefore both **31** and **32** are (like the erroneously depicted counterparts) confirmed to be incapable of modification by plasmid-borne dihydrofolate reductase. 

The name of molecule **32** has been corrected in the abstract: “Molecule **32** ({[6-(hydroxymethyl)-4-oxo-3,4,7,8-tetrahydropyrido[2,3-*d*]pyrimidin-2-yl]methylguanidinium was shown by this methodology to afford extremely stable binding towards R67 DHFR and to prevent simultaneous binding to the substrate”.

No other portions of the text dealing with molecules **31** and **32** are affected, as both the docking and the molecular dynamics simulations described therein were performed with the correct 1,2-dihydropyridine-containing structures.

The authors state that the scientific conclusions are unaffected. This correction was approved by the Academic Editor. The original publication has also been updated.

## Figures and Tables

**Table 1:** PBE0/6-311+G(2d,p)//PBE0/6-31+G(d) energies for the protonation (at position 5) and hydride transfer from NADH to each molecule (in its original protonation state). Bolded values show molecules significantly less basic than the pteridine core (>10 kcal·mol^−1^ difference) or with reduction energies above 22 kcal·mol^−1^.

Molecules	Protonation Energy (kcal/mol) (vs. Dihydrofolate Core)	Reduction by NADH (kcal/mol)	Molecule	Protonation Energy (kcal/mol) (vs. Dihydrofolate Core)	Reduction by NADH (kcal/mol)
**1**	0.0	**35.2**	**17**	**10.6**	**28.0**
**2**	4.0	**28.8**	**18**	8.2	**26.2**
**3**	2.8	**33.5**	**19**	**10.5**	**22.9**
**4**	7.0	**26.3**	**20**	**20.1**	**46.0**
**5**	9.4	22.0	**21**	**26.0**	**37.2**
**6**	−2.9	**38.4**	**22**	**25.1**	**40.2**
**7**	−9.1	**39.6**	**23**	**33.8**	**32.2**
**8**	1.8	**33.2**	**24**	7.9	4.5
**9**	−0.4	**36.3**	**25**	8.2	20.3
**10**	−2.4	**37.8**	**26**	**11.4**	2.3
**11**	**13.9**	**53.8**	**27**	**11.7**	3.6
**12**	2.2	**30.5**	**28**	**16.1**	2.2
**13**	9.8	**23.8**	**29**	8.6	16.1
**14**	9.0	5.8	**30**	**11.7**	16.2
**15**	**16.8**	**40.7**	**31**	**20.7**	**42.8**
**16**	8.7	10.4	**32**	**24.1**	**39.7**
