# Supplementary material for: Computational Development of Inhibitors of Plasmid-Borne Bacterial Dihydrofolate Reductase
Source: Antibiotics (Basel). 2022 Jun 7;11(6):779. doi: 10.3390/antibiotics11060779 (PMC9220120; doi:10.3390/antibiotics11060779)
Supplement: Supplementary file 1 [file antibiotics-1753981-supplementary.pdf › antibiotics-1753981-supplementary.pdf]

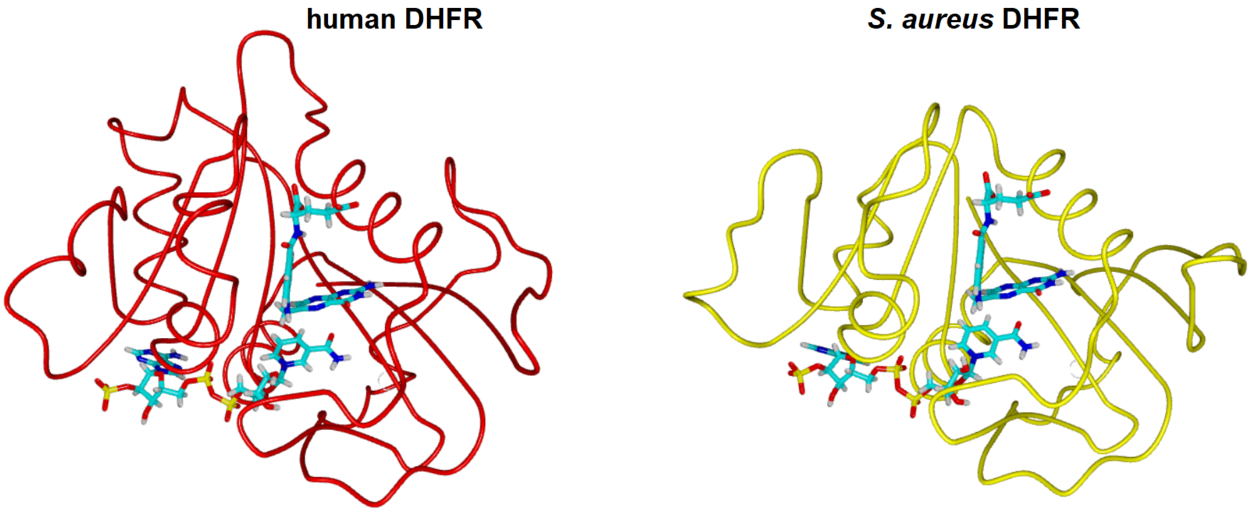


```
human      VGSLNCIVAVSQNMGIGKNGDLPWPPLRNEFRYFQRMTTTSSVEGKQNLVIMGKKTWFSIPEKNRPLKGRINLVL
S. aureus  --TLSILVAHDLQRVIGFENQLPW-HLPNDLKHVKKLSTGHT-------LVMGRKTFESI---GKPLPNRRNVVL

human      SRELKEPPQGAHFLSRSLDDALKLTEQPELANKVDMVWIVGGSSVYKEAMNHPGHLKLFVTRIMQDFESDTFFPE
S. aureus  TSDTSFNVEGVDVI-HSIEDIYQLPGH----------VFIFGGQTLFEEMIDKVD--DMYITVIEGKFRGDTFFPP

human      IDLEKYKLLPEYPGVLSDVQEEKGIKYKFEVYEKND
S. aureus  YTFEDWEVASSVEGKL---DEKNTIPHTFLHLIRK-
```

Figure S1. Structure and sequence comparison of human and *S. aureus* DHFR.
